# The Impact of Different Anesthetics on the Distribution and Cytotoxic Function of NK Cell Subpopulations: An In Vitro Study

**DOI:** 10.3390/ijms252011045

**Published:** 2024-10-14

**Authors:** Tristan J. Vulcano, Wayel H. Abdulahad, Matijs van Meurs, Rianne M. Jongman, Michel M. R. F. Struys, Dirk J. Bosch

**Affiliations:** 1 Department of Anaesthesiology, University Medical Centre Groningen (UMCG), University of Groningen, 9713 GZ Groningen, The Netherlands; t.j.vulcano@umcg.nl (T.J.V.); r.m.jongman01@umcg.nl (R.M.J.); m.m.r.f.struys@umcg.nl (M.M.R.F.S.); 2 Department of Rheumatology and Clinical Immunology, University Medical Centre Groningen (UMCG), University of Groningen, 9713 GZ Groningen, The Netherlands; w.abdulahad@umcg.nl; 3 Department of Pathology and Medical Biology, University Medical Centre Groningen (UMCG), University of Groningen, 9713 GZ Groningen, The Netherlands; m.van.meurs@umcg.nl; 4 Department of Critical Care, University Medical Centre Groningen (UMCG), University of Groningen, 9713 GZ Groningen, The Netherlands; 5 Department of Basic and Applied Medical Sciences, Ghent University, 9000 Gent, Belgium

**Keywords:** total intravenous anesthesia, volatile anesthetics, natural killer cells, immunology, lidocaine, propofol, sevoflurane

## Abstract

Only some subpopulations of natural killer (NK) cells have cytotoxic functionality, and the effects of anesthetics on these subpopulations are unknown. This study aimed to evaluate the in vitro effects of various anesthetics, both alone and in combination, on the distribution and cytotoxic function of NK cells and their subpopulations. Peripheral blood mononuclear cells (PBMCs) from eight healthy volunteers were treated for 4 h in vitro with dexmedetomidine, remifentanil, lidocaine, propofol, sevoflurane, and combinations in clinically relevant concentrations or left untreated. Flow cytometry was used to quantify the percentage of sampled NK cells and evaluate their distribution (CD56^bright^CD16^neg^, CD56^bright^CD16^dim^, CD56^dim^CD16^neg^, CD56^dim^CD16^bright^, and CD56^neg^CD16^bright^) and cytotoxicity (Granzyme B (GrzB) and perforin) of NK cell subpopulations. Although the percentage of total NK cells did not change following exposure to anesthesia, the most important cytotoxic subpopulation (CD56^dim^CD16^bright^ NK cells) decreased after exposure to both propofol (−3.58%, *p* = 0.045) and sevoflurane (−16.10%, *p* = 0.008) alone, and most combinations, especially in combination with lidocaine (propofol with lidocaine (−9.66%, *p* = 0.002) and sevoflurane with lidocaine (−21.90%, *p* < 0.001)). Dexmedetomidine and remifentanil had no effect on CD56^dim^CD16^bright^ NK cells. Furthermore, no anesthetic regimen or combination altered the expression of GrzB and perforin in NK cells or NK cell subpopulations. In short, propofol and sevoflurane suppressed the highly cytotoxic phenotype (CD56^dim^CD16^bright^) of NK cells, with those exposed to sevoflurane combinations showing greater reductions. Immunosuppression was intensified with the inclusion of lidocaine in the anesthetic regimen.

## 1. Introduction

A substantial proportion of patients with cancer undergo surgical interventions as an integral element of their therapeutic course, with the consequential challenges of cancer recurrence and metastasis significantly affecting patient outcomes. The likelihood of recurrence or metastasis subsequent to surgical resection is primarily governed by tumor-related factors, and the reduction in anti-tumor immune responses during anesthesia and surgery is postulated to be involved [1]. Numerous publications have also elucidated a compelling association between the *type* of anesthesia and the multifaceted dynamics of this immune response. Recently, various meta-analyses have suggested that total intravenous anesthesia (TIVA) with propofol has a protective effect on immune function, accompanied by an overall improvement in patient survival [2,3,4]. In addition, other studies have shown that the commonly used volatile anesthetics isoflurane and sevoflurane attenuate natural killer (NK) cell tumor cytotoxicity [5,6], whereas TIVA has been proposed to either have no effect [7,8] or increase NK cell numbers and cytokine secretion [9]. 

NK cells are vital to the anti-cancer immune response, as they possess the ability to recognize and lyse tumor cells without prior exposure to tumor antigens [10,11]. Human NK cells display distinct subpopulations distinguished by varying expression levels of the NK cell adhesion molecule CD56 and activating receptor CD16 (Table 1). The two major NK subpopulations include CD56^bright^CD16^dim/neg^ NK cells, traditionally identified as immature precursors with cytokine-producing capability, and the CD56^dim^CD16^bright^ subpopulation, acknowledged for its heightened cytotoxicity and release of granzyme-B (GrzB) and perforin [12], which mediate their tumor-killing ability [13]. 

Limited data are available on the effect of anesthetics on functionally distinct NK cell subpopulations, which could be useful in refining our knowledge of NK cell functionality. Therefore, our aim was to study the effect of different commonly used anesthetics alone (dexmedetomidine, remifentanil, lidocaine, propofol, and sevoflurane) and in combination on NK cell proliferation and cytotoxicity, regarding both total NK cells and relevant NK cell subpopulations. 

## 2. Results

### 2.1. Unaltered NK Cell Percentages and Cytotoxicity upon Anesthetic Exposure

We first assessed the percentage of *total* NK cells within the lymphocyte population following exposure to each anesthetic, including dexmedetomidine, remifentanil, lidocaine, propofol, sevoflurane, and combinations. One sample exposed to sevoflurane with lidocaine was excluded from all analyses owing to a technical error. The percentage of *total* NK cells did not change after exposure to any anesthetic or combination (Supplemental Data: Figure S1B). 

Next, we assessed the impact on the cytotoxic function of NK cells by analyzing the expression of GrzB and perforin within the total NK cell population after exposure to each anesthetic vs. the control condition. No differences were found in the expression of either GrzB or perforin or their combined expression in any of the combinations, upon exposure to various anesthetics or combinations (Supplemental Data: Figure S2B). 

### 2.2. Differential Effects of Anesthetics on the Distribution of NK Cell Subpopulations

**Figure 1:**
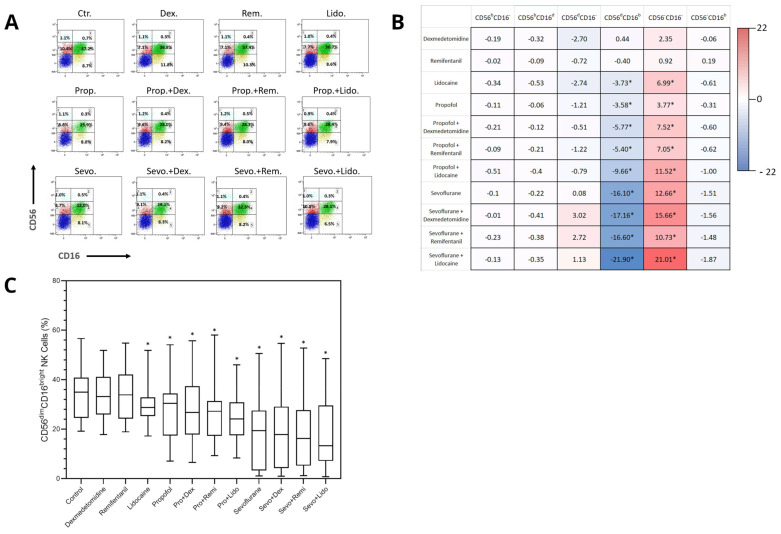
Effects of dexmedetomidine, remifentanil, lidocaine, propofol, and sevoflurane as single drugs and in clinically relevant combinations on CD56 and CD16 expression in natural killer (NK) cells. Peripheral blood mononuclear cells were exposed to dexmedetomidine, remifentanil, lidocaine, propofol, and sevoflurane. Data are presented as percentage change in the median of 8 independent samples compared to control. (**A**) FACS plot outlining the distribution of NK cell subpopulations based on the relative expression of CD56 and CD16, and accordingly subdivided into CD56^bright^CD16^neg^ (light blue; 1), CD56^bright^CD16^dim^ (light green; 2), CD56^dim^CD16^neg^ (red; 3), CD56^dim^CD16^bright^ (green; 4), and CD56^neg^CD16^bright^ (yellow; 5) subpopulations, and CD56^neg^CD16^neg^ (blue). (**B**) Percentage change in CD56 and CD16 expression to control in NK cell subpopulations; bright (b) and dim (d). (**C**) Distribution of CD56^dim^CD16^bright^ NK cell frequency after each treatment. Benjamini–Hochberg-adjusted *p*-values (q): * q < 0.05.

NK cell subpopulations exhibited a distinct distribution following exposure to various anesthetics. The CD56^dim^CD16^bright^ NK subpopulation, which is crucial for anti-tumor cytotoxicity, was reduced after exposure to lidocaine (−3.73%, *p* = 0.024), propofol (−3.58%, *p* = 0.045), and sevoflurane alone (−16.10%, *p* = 0.008). Additionally, combinations of propofol with dexmedetomidine (−5.77%, *p* = 0.044), propofol with remifentanil (−5.40%, *p* = 0.012), propofol with lidocaine (−9.66%, *p* = 0.002), sevoflurane with dexmedetomidine (−17.16%, *p* = 0.005), sevoflurane with remifentanil (−16.60%, *p* = 0.002), and sevoflurane with lidocaine (−21.90%, *p* < 0.001) demonstrated significant reductions in the CD56^dim^CD16^bright^ NK subpopulation (Figure 1C). 

Comparing each treatment with propofol to its sevoflurane counterpart, all combinations with sevoflurane showed a significantly greater decrease in CD56^dim^CD16^bright^ cells, including propofol with dexmedetomidine vs. sevoflurane with dexmedetomidine (−6.29%, *p* = 0.04), with remifentanil (−6.72%, *p* = 0.031), and with lidocaine (−8.13%, *p* = 0.021). The difference between propofol alone vs. sevoflurane alone was not statistically significant. A similar pattern was observed in the subpopulation of CD56^neg^CD16^neg^ cells following anesthetic exposure, exhibiting a relative percentage increase with exposure to lidocaine (6.99%, *p* = 0.008), propofol (3.77%, *p* = 0.012), and sevoflurane alone (12.66%, *p* = 0.015). Furthermore, combinations of propofol with dexmedetomidine (7.52%, *p* = 0.035), propofol with remifentanil (7.05%, *p* = 0.007), propofol with lidocaine (11.52%, *p* < 0.001), sevoflurane with dexmedetomidine (15.66%, *p* = 0.01), sevoflurane with remifentanil (10.73%, *p* = 0.005), and sevoflurane with lidocaine (21.01%, *p* < 0.001) demonstrated a significant percentage increase in CD56^neg^CD16^neg^ cells (Figure 1B). There was no significant change in the percentage of cell distribution in any other NK cell subpopulation.

### 2.3. Anaesthesia Exposure Does Not Affect the Cytotoxicity of NK Cell Subpopulations

The cytotoxic function of each NK cell subpopulation was evaluated by determining the percentage of intracellular GrzB and perforin expression (Supplemental Figure S3B–F). No differences were found in the expression of either GrzB or perforin or their combined expression in any NK cell subpopulation or CD56^neg^CD16^neg^ cells after exposure to each anesthesia and combination.

## 3. Discussion

With this study, we have shown that commonly used anesthetics and their combinations have a profound effect on the distribution of NK cell subpopulations. An important finding of this study is that the percentage of the *total* NK cell population did not change following exposure to anesthesia; however, after examining different NK cell subpopulations, a significant decrease in the frequency of the highly cytotoxic CD56^dim^CD16^bright^ subpopulation was found for anesthetic regimens containing propofol or sevoflurane. The reduction in regimens containing sevoflurane anesthesia showed a greater decrease than that after propofol anesthesia combinations. Finally, lidocaine appears to amplify the inhibitory effect, causing a decrease in the same highly cytotoxic subpopulation when applied alone and inducing the largest decline in distribution when combined with propofol (9.66%) and sevoflurane (21.90%). 

The differences between inhalational volatile anesthetics (IHVA) and total intravenous anesthesia (TIVA) have been the primary focus of anesthesia-related immune modulation, although the findings have been conflicting. Numerous randomized control trials have found that IHVAs have a suppressive effect on NK cell cytotoxicity, whereas propofol (TIVA) is assumed to have a protective effect [3,4,7,9,14,15]. In contrast, other studies have found no differences between the two anesthetic modalities in terms of various biomedical and patient outcome measures. For example, no differences in NK cell count, cytotoxic T lymphocyte (CTL) count, or apoptosis rate of tumor cells were observed when blood from breast cancer surgical patients were exposed to propofol or sevoflurane [8]. Furthermore, no difference in the fraction of NK cells or T lymphocytes was observed when these anesthetics were administered to surgical patients with colorectal cancer [16]. Similarly, in the current study, no significant change was observed in the frequency of total sampled NK cells after exposure to each anesthetic and combination. However, after dividing NK cells into functional subpopulations, CD56^dim^CD16^bright^ NK cells exhibited a significant decrease in percentage when exposed to any regimen containing either propofol or sevoflurane. It should be noted that each sevoflurane combination resulted in a greater percentage decrease (ranging from 17.16% to 21.9%) in the CD56^dim^CD16^bright^ NK cell subpopulation compared to its propofol counterpart. This decrease in percentage was relative to the other cells gated for the analysis, as surface CD markers and lytic molecule expression were measured using percentage differences between NK cell phenotypes. These cells cannot further proliferate in vitro, and changes in the frequency of a given NK cell subpopulation are due to a shift from another NK cell subpopulation(s). Following this, there should be a relative percentage increase in any number of other NK cell subpopulations. Interestingly, there was only a significant, subsequent increase in the percentage of CD56^neg^CD16^neg^ cells with the same pattern as the decreased CD56^dim^CD16^bright^ NK cell subpopulation (Figure 1B). The cell population lacking the expression of both CD56 and CD16 (CD56^neg^CD16^neg^) is functionally not classified as NK cells and the increase in their percentage points to the likelihood that NK cells are stripped of their CD56 and CD16 surface markers after exposure to anesthesia. This entails losing homotypic adhesion and antibody-mediated immunity capabilities, respectively, and could lead to reduced cell lytic function. This should be further researched in more detail, as there was no observed difference in GrzB or perforin expression between any NK cell subpopulation or in CD56^neg^CD16^neg^ cells exposed to propofol, sevoflurane, or combinations. 

The synthetic opioid remifentanil has been shown to decrease NK cell activity and T cell proliferation in a rat model [17] and has been shown to have no effect on NK cells when infused for 8 h in healthy human subjects [18]. Moreover, dexmedetomidine, a selective alpha2-adrenoceptor agonist, has been shown to decrease perioperative stress hormones, decrease markers of inflammation, and increase the expression of NK, T, and B cells [19]. These protective effects were not observed in our findings and these analgesics did not alter the immunosuppressive effects of propofol or sevoflurane, but future research should clarify whether adjuvant use of dexmedetomidine or remifentanil can significantly attenuate perioperative immunosuppression by anesthetic agents. 

Other important results were obtained with lidocaine. When studying the effects of local anesthetics on cell-mediated immunity, lidocaine increased NK cell cytotoxicity at clinically relevant concentrations [20], with Ramirez and colleagues [21] showing this enhanced activity was mediated through GrzB. This enhancement in NK cell cytotoxicity was not observed in the present study. Lidocaine alone significantly reduced the percentage of the CD56^dim^CD16^bright^ NK cell subpopulation (3.73%) while subsequently increasing the CD56^neg^CD16^neg^ subpopulation and strengthening the suppressive effects of propofol (9.66%) and sevoflurane (21.90%) on the CD56^dim^CD16^bright^ NK cells. This unprecedented immunosuppressive effect of lidocaine has likely not been observed when NK cells are studied as a complete population. 

There are limitations to note for the current study. Importantly, the study was conducted using a relatively small number of blood samples from healthy volunteers without a cancer history, and exposure to anesthetic agents was performed in vitro. By examining samples from healthy volunteers, we were able to explore our aim of analyzing the impact of anesthetics, both individually and in combination, on NK cell subpopulations without heterogeneity of different cancer subtypes or treatment histories. The current study design may further be used as a framework for future research using samples obtained from oncology patients, offering a more comprehensive understanding of the clinical relevance of these findings. Likewise, although there are numerous benefits to performing in vitro analyses, it is not possible to capture the complex mechanisms and interactions present within in vivo human subjects. Therefore, although this study has built on the current body of evidence by investigating anesthetic effects on NK cell subpopulations, future studies should approximate the in vivo environment more closely. Additionally, from the data collected for this study, we were unable to determine the exact change in the number of cells in each NK cell subpopulation following treatment, because the population of each subpopulation was measured as a percentage of the total cells. It is therefore ambiguous whether the increase in the CD56^neg^CD16^neg^ subpopulation is due to other subpopulations losing CD56 or CD16. Finally, no differences were observed in GrzB and perforin expression in any given NK cell population. However, the results may demonstrate whether NK cells are able to modulate their expression of tumor-lytic molecules in vitro. Based on this study, it seems that GrzB and perforin expression are, at least, not altered by exposure to anesthesia alone, and would likely require the involvement of more complex in vivo mechanisms. 

## 4. Materials and Methods

### 4.1. Peripheral Blood Mononuclear Cells Isolation

Lithium-heparinized venous blood was obtained from eight healthy donors (aged 29–55 years, 4 ♂ and 4 ♀, all without medical history). According to the Medical Ethical Review Board of the University Medical Center of Groningen (Chairperson Prof W.A. Kamps) on 17 December 2019, no WMO approval was required (METc 2019/655). Peripheral blood mononuclear cells (PBMCs) were immediately isolated by density-gradient centrifugation using Lymphoprep (Stemcell Technologies, Cambridge, UK). PBMCs were next washed with phosphate-buffered saline (PBS) and resuspended at 1 × 10^6^ cells/mL in RPMI 1640 (Cambrex Bio Science, Verciers, Belgium) supplemented with 5% fetal calf serum (FCS) and 50 mg/mL gentamycin (Gibco, Scotland, UK). 

### 4.2. Exposure of Cells to Anesthetic Agents

PBMCs were aliquoted into eight separate polypropylene tubes (1 × 10^6^ cells per tube) and treated with either dexmedetomidine (10 ng/mL), remifentanil (10 ng/mL), lidocaine (4 µg/mL), propofol (10 µg/mL), or the combined agents (propofol + dexmedetomidine, propofol + remifentanil, or propofol + lidocaine). One tube was left untreated to serve as the control sample. The samples were then incubated for 4 h at 37 °C with 5% CO_2_. 

PBMCs were seeded in a 12-well culture plate (1 × 10^6^ cells per well) and exposed to sevoflurane in the operating room. The culture plate was placed in an air-tight plastic bag and connected to a mechanical ventilator (Zeus ventilator, Dräger, Lübeck, Germany) via a tube equipped with breathing ports. Sevoflurane was administered through a vaporizer and end-tidal sevoflurane concentrations were measured. Cells were exposed to sevoflurane 2% alone or in combinations (sevoflurane + dexmedetomidine, sevoflurane + remifentanil, or sevoflurane + lidocaine) for 4 h and placed under a heat blanket maintained at 38 °C. 

The concentrations used were clinically relevant, and the chosen anesthetics all have their own mechanisms of action. We chose to investigate both propofol (TIVA) and sevoflurane (IHVA), as well as a commonly used opiate (remifentanil), a stress-reducing alpha2-adrenoceptor agonist (dexmedetomidine), and a local anesthetic (lidocaine). In addition, the chosen combinations were clinically relevant, and the duration of exposure to anesthetics was equal to the average operating time.

### 4.3. Flow Cytometry Analysis of NK Cells

After incubation with anesthetic agents, the cells were washed with RPMI + 5%FCS, and Brefaldin A (BFA, 10 µg/mL) was added to each sample to inhibit the release of cytotoxic proteins from NK cells. The cells were then incubated for 16 h at 37 °C with 5% CO_2_.

Following incubation, cells were labeled with the cell surface markers anti-human CD3 PerCP (BD Biosciences, San Jose, CA, USA), anti-human CD19 Brilliant Violet 605 (BD Biosciences), anti-human CD16 BUV (BD Biosciences), and anti-human CD56 Brilliant Violet 785 (BioLegend, San Diego, CA, USA) for 15 min at room temperature. Next, the cells were fixed with 100 µL of fixation reagent A (Fix/Perm medium A, Thermo Fisher Scientific, Waltham, MA, USA) for 15 min at room temperature in the dark. After washing, the cells were resuspended in 100 µL of permeabilization reagent B (Fix/Perm medium B, Thermo Fisher Scientific) and labeled with anti-human GrzB Alexa647 (BD Biosciences) and anti-human Perforin Brilliant Violet 421 (BD Biosciences) for 30 min at room temperature in the dark. The cells were washed and analyzed using an LSR-II flow cytometer (BD). Data were collected for 1 × 10^6^ events per sample and plotted using the Kaluza software package version 2.0.0 (Beckman Coulter, Brea, CA, USA). After excluding B cells (CD19) and T cells (CD3), NK cells (CD19^neg^CD3^neg^) were gated and categorized based on the relative expression of CD56 and CD16 on their surface and accordingly subdivided into CD56^bright^CD16^neg^, CD56^bright^CD16^dim^, CD56^dim^CD16^neg^, CD56^dim^CD16^bright^, and CD56^neg^CD16^bright^ subpopulations (Figure 1A). The intracellular expression of GrzB and Perforin was assessed in total and in each NK cell subpopulation.

### 4.4. Statistical Analysis

All data were analyzed using Statistical Package for Social Sciences (SPSS) version 28.0.0 (SPSS Inc., Chicago, IL, USA). There were no outliers, as assessed by the examination of studentized residuals for values greater than ±3. Data were tested for normal distribution using Shapiro–Wilk’s test of normality on the studentized residuals (*p* > 0.05). For normalized data, differences in percentages compared to the control were analyzed using one-way analysis of variance (ANOVA), and post-hoc analyses were performed using Dunnett’s correction. For non-normalized data, differences were analyzed using the Friedman test, and post-hoc analyses were performed using Wilcoxon signed-rank tests. The Benjamini–Hochberg (BH) procedure was used to correct for multiple comparisons, and *p*-values under the BH critical value were considered significant. Original, unadjusted *p*-values that were significant after the BH procedure are presented. Differences in percentages compared to the control or between treatments are presented as medians for total NK cells and each NK cell phenotype and for GrzB and Perf expression in each NK cell population.

## 5. Conclusions

The proportion of total NK cells did not change after in vitro exposure to anesthesia. However, after subdividing NK cells into subpopulations, both propofol and sevoflurane caused a decrease in the percentage of the highly cytotoxic CD56^dim^CD16^bright^ NK cell subpopulation, with the effect of sevoflurane appearing greater than that of propofol. Furthermore, the combination of lidocaine with propofol or sevoflurane caused a greater decrease in the CD56^dim^CD16^bright^ NK cell subpopulation. Therefore, the choice of anesthesia appears to influence the cytotoxic function of NK cells and might influence oncological survival. Based on these results, we would advise the use of propofol anesthesia in combination with an opioid during oncologic surgery. It should be noted, however, that our results were not obtained in an oncologic patient population and that in vivo studies are needed to confirm our results.

## Figures and Tables

**Table 1:** Relevant functionality of natural killer subpopulations distinguished by surface expression of CD56 and CD16. + present; − absent; ++ present to a significant extent.

	Antibody-Mediated Immunity	Proliferation Ability	Cytokine Production	Cytolytic Molecule Production
CD56^bright^CD16^neg^	−	++	++	−
CD56^bright^CD16^dim^	+	++	++	−
CD56^dim^CD16^neg^	−	+	+	++
CD56^dim^CD16^bright^	++	+	+	++
CD56^neg^CD16^bright^	++	+	−	−

## Data Availability

The raw data supporting the conclusions of this article will be made available by the authors on request.

## Supplementary Materials

The following supporting information can be downloaded at: https://www.mdpi.com/article/10.3390/ijms252011045/s1.
