# Supplementary material for: The Impact of Different Anesthetics on the Distribution and Cytotoxic Function of NK Cell Subpopulations: An In Vitro Study
Source: Int J Mol Sci. 2024 Oct 14;25(20):11045. doi: 10.3390/ijms252011045 (PMC11507532; doi:10.3390/ijms252011045)
Supplement: Supplementary file 1 [file ijms-25-11045-s001.zip › ijms-3214074-supplementary.pdf]

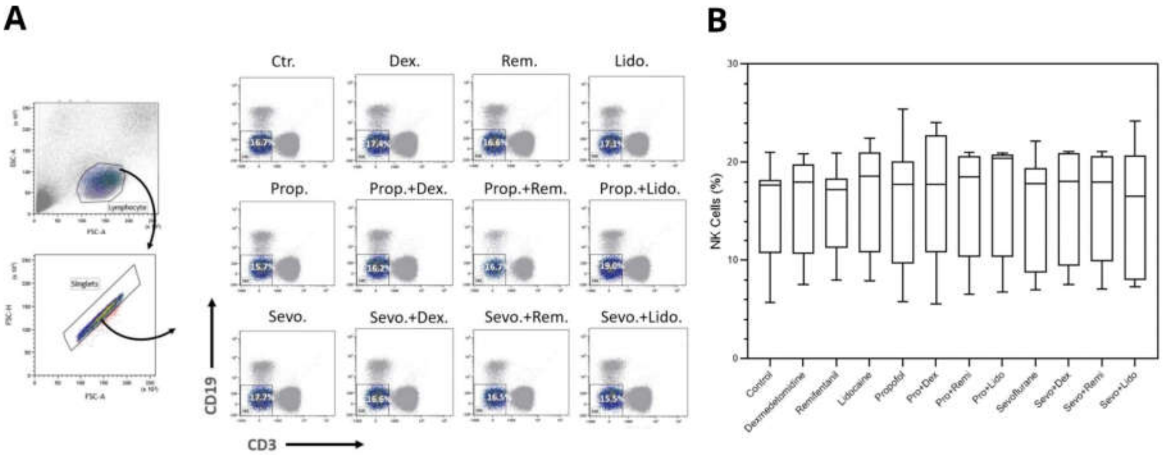

Figure S1. Effects of dexmedetomidine, remifentanil, lidocaine, propofol, and sevoflurane as single drugs and in clinically relevant combinations on total natural killer cells. PBMCs were exposed to dexmedetomidine, remifentanil, lidocaine, propofol, and sevoflurane. Data are presented as percentage change of the median of 8 independent samples to control. A. Representative FACS plots and gating strategy identifying total NK cells. Peripheral blood lymphocytes were identified and gated by their forward (FSC) and side (SSC) scatter and doublets were excluded in the FSC-H/FSC-A plot. After excluding B cells (CD19) and T cells (CD3), percentage of total NK cells ($CD19^{neg}CD3^{neg}$) were determined after exposure to each anaesthesia and the control condition. B. Distribution of total NK cell frequency after each treatment.

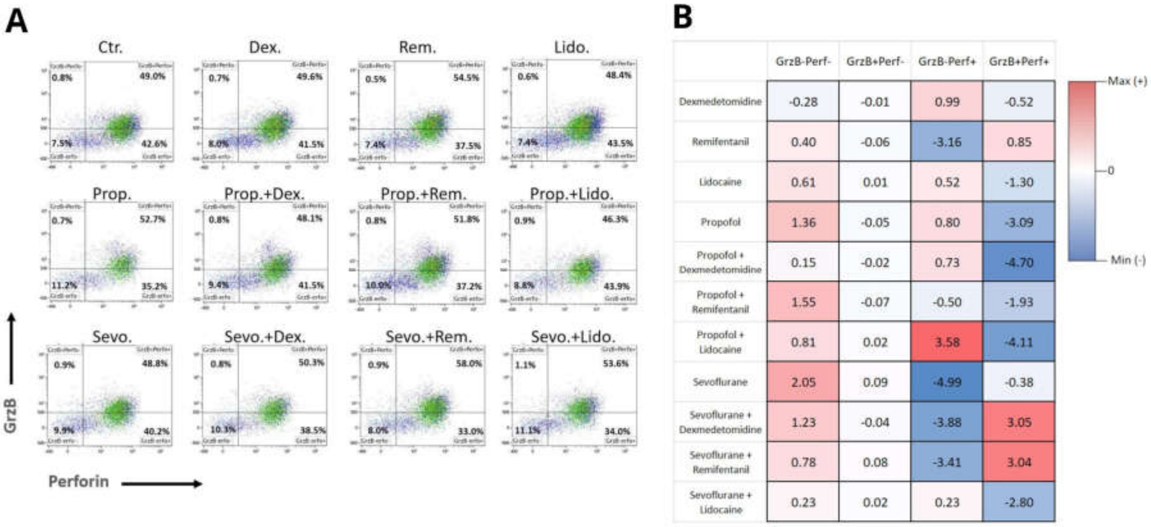

	GrzB-Perf-	GrzB+Perf-	GrzB-Perf+	GrzB+Perf+
Dexmedetomidine	-0.28	-0.01	0.99	-0.52
Remifentanil	0.40	-0.06	-3.16	0.85
Lidocaine	0.61	0.01	0.52	-1.30
Propofol	1.36	-0.05	0.80	-3.09
Propofol + Dexmedetomidine	0.15	-0.02	0.73	-4.70
Propofol + Remifentanil	1.55	-0.07	-0.50	-1.93
Propofol + Lidocaine	0.81	0.02	3.58	-4.11
Sevoflurane	2.05	0.09	-4.99	-0.38
Sevoflurane + Dexmedetomidine	1.23	-0.04	-3.88	3.05
Sevoflurane + Remifentanil	0.78	0.08	-3.41	3.04
Sevoflurane + Lidocaine	0.23	0.02	0.23	-2.80

Figure S2. Effect of dexmedetomidine, remifentanil, lidocaine, propofol, and sevoflurane as single drugs and in clinically relevant combinations on functional cytotoxicity of total natural killer cells. PBMCs were exposed to dexmedetomidine, remifentanil, lidocaine, propofol, and sevoflurane. Data are presented as percentage change of the median of 8 independent samples to control. C. FACS plot outlining the expression of GrzB and Perf in total NK cells after each treatment. D. Percentage difference in expression of GrzB and Perf to control in total NK cells.

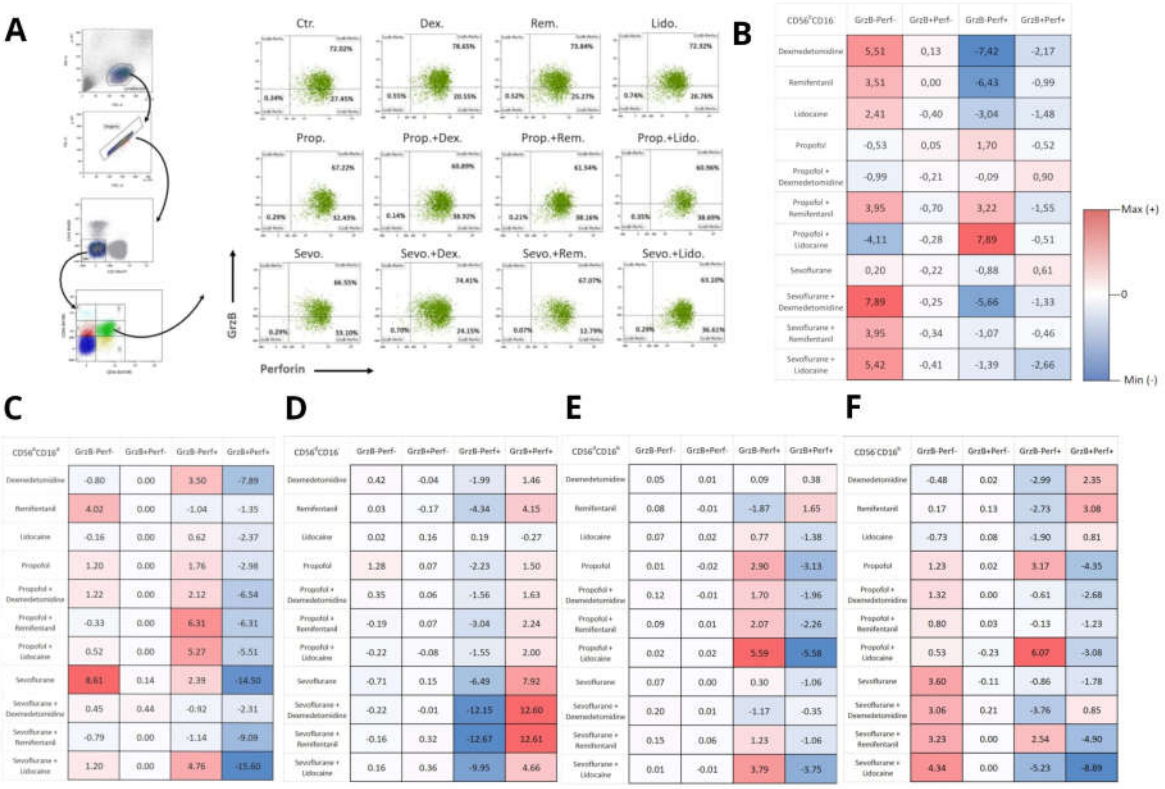

Figure S3. Effect of dexmedetomidine, remifentanil, lidocaine, propofol, and sevoflurane as single drugs and in clinically relevant combinations on functional cytotoxicity of natural killer cells divided into CD56 and CD16 subsets. PBMCs were exposed to dexmedetomidine, remifentanil, lidocaine, propofol, and sevoflurane. Data are presented as percentage change of the median of 8 independent samples to control. A. FACS plot outlining the gating strategy of natural killer cells. Peripheral blood lymphocytes were identified and gated by their forward (FSC) and side (SSC) scatter and doublets were excluded in the FSC-H/FSC-A plot. After excluding B cells (CD19) and T cells (CD3), NK cells ($CD19^{neg}CD3^{neg}$) were selected and subclassified into five subsets according to their surface expression of CD56 and CD16 markers. Each NK subset was then analyzed for the expression of GrzB and Perf after each treatment. **B-F.** Percentage change in different NK cell subsets to control. $CD56^{bright}CD16^{neg}$ (B), $CD56^{bright}CD16^{dim}$ (C), $CD56^{dim}CD16^{neg}$ (D); $CD56^{dim}CD16^{bright}$ (E), $CD56^{neg}CD16^{bright}$ (F). Data presented as percentage changes of the median of each independent sample to its own control.
